# The Human Myometrial Transcriptome and the DNA Methylome of Testosterone-treated Patients Resemble the Myometria from Fibroid Patients

**DOI:** 10.1007/s43032-025-01893-9

**Published:** 2025-06-05

**Authors:** Emmanuel N. Paul, Tyler J. Carpenter, Andrew Bossick, Ghassan Allo, Ganesa R. Wegienka, Jose M. Teixeira

**Affiliations:** 1 Department of Obstetrics, Gynecology and Reproductive Biology, College of Human Medicine, Michigan State University, Grand Rapids, MI 49503 USA; 2 Department of Public Health Sciences, Henry Ford Health, Detroit, MI 48202 USA; 3 Department of Pathology, Henry Ford Hospital, Detroit, MI 48202 USA

**Keywords:** Leiomyoma, Myometrium, Testosterone, Transcriptome, Methylome

## Abstract

Uterine fibroids, or leiomyomas, are noncancerous tumors of the myometrium and the most common tumors in women, with a cumulative incidence of approximately 80% by age 50. Currently, hysterectomy is the only definitive cure, and effective non-hormonal therapeutics are lacking. Understanding the etiology of fibroids may lead to alternative, less invasive treatments. Several obstetric disorders, including polycystic ovary syndrome (PCOS), have been linked to uterine fibroids, and women with PCOS often exhibit hormonal imbalances, particularly elevated serum testosterone levels. However, the impact of testosterone on the myometrium remains poorly understood. We hypothesize that elevated testosterone may increase the risk of developing uterine fibroids. Using RNA sequencing and MethylationEPIC array analyses, we compared myometrial tissue from women without fibroids (MyoN, n = 33), with fibroids (MyoF, n = 66), and after testosterone therapy as part of clinical care for gender dysphoria (MyoT, n = 7). The transcriptomic and methylation profiles of MyoT clustered with MyoF and were distinct from MyoN. We identified 1,321 differentially expressed protein-coding genes between MyoT and MyoN, while only 494 were found between MyoT and MyoF. Disease ontology analysis of MyoT vs. MyoN revealed enrichment of the fibroid tumor gene set. Fibroid associated genes including *TGFβ3*, *CCND1*, *SERPINE1,* and *FGFR1* were upregulated in MyoT and MyoF samples compared to MyoN samples. The DNA methylation profiles of MyoT were closer to those of MyoF, but no correlation was observed between methylation status and gene expression. Our preliminary data suggest that exogenous testosterone induces transcriptional and methylation changes in the myometrium consistent with those observed in MyoF tissues. These findings suggest that elevated testosterone may be associated with an increased risk of developing uterine fibroids.

**Supplementary Information:**

The online version contains supplementary material available at 10.1007/s43032-025-01893-9.

## Introduction

Uterine fibroids, or uterine leiomyomas, are noncancerous solid neoplasms that arise from the smooth muscle cells of the myometrium [1]. They are the most common tumors of the female reproductive tract, with a cumulative incidence of approximately 80% by age 50 that varies varying across different racial and ethnic groups [2–4]. Black women are at greater risk, experiencing fibroids at younger ages and with more severe symptoms [5, 6]. Symptomatic fibroids can cause heavy menstrual bleeding, anemia, pelvic pain, and dysmenorrhea, significantly affecting a patient's quality of life [7]. Currently, hysterectomy is the only definitive cure for symptomatic fibroids, and accounts for approximately 50% of all hysterectomies performed in the United States [8]. Despite the effectiveness of hysterectomy, it is an invasive solution that ends fertility, underscoring the need for less invasive treatments. Non-surgical options, such as hormonal therapies, can provide temporary relief, but long-term use is not approved because of associated side effects [9].

The etiology of uterine fibroids is thought to be multifactorial, involving genetic, hormonal, and environmental factors [10]. While estrogen and progesterone are well-established drivers of fibroid growth [11, 12], the role of androgens, particularly testosterone, is less understood. Patients with polycystic ovary syndrome (PCOS), who often exhibit elevated testosterone levels, have been shown to be at higher risk for fibroids [13], yet the specific effects of testosterone on the myometrium remain unclear.

Our previous studies demonstrated that the myometrium from fibroid patients (MyoF) differs significantly in its transcriptomic profile compared with myometrium from non-fibroid patients (MyoN), suggesting that the myometrium itself may actively contribute to fibroid pathogenesis [14]. Additionally, we, along with others, have recently published that racial disparities are also observed in myometrial biology [15, 16], with the myometrium from Black fibroid patients more closely resembling fibroid tissue than the myometrium from White fibroid patients. These differences in Black patients'myometria could help explain the higher incidence and severity of fibroids in Black individuals, pointing to a potential role of myometrial biology in the racial disparity observed in fibroid burden. This highlights the importance of studying how endogenous and exogenous factors, including steroid hormones, affect myometrial function. In this current study, we provide evidence to support our hypothesis that testosterone induces transcriptomic changes in the myometrium that drive it toward a more MyoF phenotype. By examining whether testosterone therapy alters the myometrial transcriptome to resemble that of fibroid patients, we aim to shed light on the mechanisms by which environmental exposures to endocrine disrupting compounds contribute to fibroid development.

## Materials and Methods

### Sample Collection

The use of human tissue specimens was approved by Michigan State University and Spectrum/Corewell Health’s Institutional Review Boards (IRB) as secondary use of biobank or study materials without associated identifying patient information collected from patients who provided informed consent. Henry Ford Health’s IRB approved a protocol in which informed consent was obtained from hysterectomy patients prior to surgery to permit collection and use of human tissue specimens with associated patient information. Patient details are shown in Supplementary Table 1. Human samples were processed as previously described [14, 15]. Myometrial samples from premenopausal patients without fibroids (MyoN, n = 33), premenopausal patients with fibroids containing *MED12* mutations [17] patients (MyoF, n = 66), and patients undergoing testosterone treatment for gender dysphoria (MyoT, n = 7) were obtained following total hysterectomy from pre-menopausal (aged 21–52). No fibroids were detected by ultrasound prior to surgery for both the MyoN and MyoT samples. Gross pathology reports were provided for the patients treated with testosterone (MyoT). No abnormalities—such as adenomyosis or fibroids—were detected in the MyoT samples. Briefly, samples were washed with phosphate-buffered saline, minced, and immersed in RNAlater (MilliporeSigma) or flash frozen and stored at 80 °C for RNA-Seq analyses. The remaining tissue pieces were immediately flash frozen and stored at − 80 °C for methylation analysis. *MED12* mutation in the fibroids was determined by PCR amplification, followed by Sanger sequencing, as described in our previous study [14].

### RNA Isolation, Library Preparation and Sequencing

Tissues were homogenized in TRIzol reagent (Thermo Fischer Scientific, Fairlawn, NJ), and RNA was isolated following the manufacturer’s instructionsand stored at −80ºC in nuclease-free water. RNA integrity values from tissues were determined with an Agilent 2100 Bioanalyzer (ThermoFisher), and values ≥ 7.5 were used for library preparation and paired-end RNA-sequencing on an Illumina NextSeq sequencer. Libraries were prepared using a Kapa RNA HyperPrep kit with ribosomal reduction, pooled, and sequenced on flowcells to yield approximately 50–60 million reads/sample. Raw fastq files were deposited in the NCBI Gene Expression Omnibus (GSE288971).

### RNA-seq Analysis

RNA-seq reads were trimmed for quality and adapters using TrimGalore (version 0.6.4), and the quality of the trimmed reads waser assessed with FastQC (version 0.11.7). Trimmed reads were mapped to Homo sapiens genome assembly GRCh38 (hg38) using STAR (version 2.6.0c). Reads overlapping Ensembl annotations (version 99) were quantified with STAR prior to model-based differential expression analysis using the edgeR-robust method. Genes with low counts per million (CPM) were removed using the filterByExpr function from edgeR [18].

Uniform Manifold Approximation and Projection (UMAP) plots were generated after batch correction with the umap R package (version 0.2.10.0) to verify sample separation. Consensus clustering plots were made using the median-centered, normalized counts for the 5,000 most variable genes based on median absolute deviation and the ConsensusClusterPlus package (version 1.68.0) [19] with the parameters “reps = 1000”, “pItem = 0.8”, “pFeature = 1”, and “distance = pearson”. Briefly, these settings resample the data 1,000 times using 80% of the samples and 100% of the features each time; they then find the consensus clustering based on hierarchical clustering of each resampling using (1-Pearson correlation) as distance. Scatterplots of Principal Component (PC)1 and PC2 were constructed with the “pca” function of the PCAtools R package (version 2.16.0) to verify sample separation prior to statistical testing. Generalized linear models were used to determine if principal components were significantly associated with tissue type. Differential expression was calculated by comparing MyoF or MyoT to the control tissues MyoN, and differentially expressed genes (DEGs) were visualized with a volcano plot generated using the EnhancedVolcano (version 1.22.0) package in R. Venn diagrams of the overlapping DEGs of the tissues (MyoF vs. MyoN and MyoT vs. MyoN) were constructed using the eulerr package (version 7.0.2). Gene set enrichment and overrepresentation analyses were completed with clusterProfiler package (version 4.8.2) using MSigDB [20] and DOSE [21] reference databases. The top enriched disease gene sets associated with the female reproductive track were shown in the figure.

### DNA Isolation and DNA Methylation Analysis

DNA was isolated from snap-frozen myometrium and fibroid tissue samples, hybridized to the Infinium MethylationEPIC array (versions I and II), and analyzed essentially as previously described [22]. Raw IDAT files were deposited in NCBI GEO (GSE296589). Briefly, raw IDAT files were processed using R package SeSAMe (version 1.23.6) and the openSesame pipeline with noob background correction, non-linear dye bias correction, and non-detection masking [23, 24]. DNA methylation was measured in beta values for each probe calculated as a quantitative percentage of methylated signals over both methylated and unmethylated probe signals. Cellular composition of the samples was determined by promoter methylation of *MIR200 C/141* [25] and *ACTA2* [26] (Supplementary Fig. 2A). UMAP plot of CpG probes was performed after batch correction with the R package *ChAMP* (Version 2.36.0) using the *umap* R package (version 0.2.10.0). Differentially methylated loci (DMLs) were called on MyoF or MyoT to the control tissues MyoN using batch corrected betas in the R package SeSAMe [23]. Significant DMLs (p ≤ 0.05) were split by methylation status into hypermethylated (> 0.1) or hypomethylated (< −0.1). Venn diagrams of the overlapping DMLs of the tissues (MyoF vs. MyoN and MyoT vs. MyoN) were constructed using the *venn* package (version 1.12) and visualized using the eulerr package (version 7.0.2). Probe annotation of Illumina EPIC array (human reference genome (NCBI build GRCh38/hg38) was downloaded from [24]. Features from the annotated DMLs were used to construct barplots using *ggplot* (version 3.5.1).

### Quantitative Real Time PCR

cDNA was synthesized using the iScript cDNA Synthesis Kit (BioRad, Hercules, CA) with 1 µg of the total RNA input for confirmation of the RNA-seq results. Quantitative Real time PCR (qRT-PCR) analysis using SYBRGreen (BioRad, Hercules, CA) was performed with 7 random MyoF and MyoN samples and all 7 MyoT samples to determine relative gene expression using the CFX384 qRT-PCR System (BioRad, Hercules, CA). RPL17 was used as a reference gene for data normalization. Primer sequences used for qRT-PCR (5′−3′) were as follows;

*RPL17* forward (ACGAAAAGCCACGAAGTATCTG),

*RPL17* reverse (GACCTTGTGTCCAGCCCCAT),

*TGFB3* forward (CTAAGCGGAATGAGCAGAGGATC),

*TGFB3* reverse (TCTCAACAGCCACTCACGCACA),

*FGFR1* forward (CCCGTAGCTCCATATTGGACA),

*FGFR1* reverse (TTTGCCATTTTTCAACCAGCG),

*SERPINE1* forward (ACCGCAACGTGGTTTTCTCA),

*SERPINE1* reverse (TTGAATCCCATAGCTGCTTGAAT),

*FKBP5* forward (CTCCCTAAAATTCCCTCGAATGC),

*FKBP5* reverse (CCCTCTCCTTTCCGTTTGGTT).

### Statistics and Reproducibility

Statistical analyses were performed using the listed packages in R (version 4.4.1) described above in the RNA-seq Analysis section. DEGs in the RNA-seq of MyoF vs. MyoN, MyoT vs. MyoF and MyoT vs. MyoN were identified as those having a Benjamini–Hochberg FDR corrected p < 0.05 [27]. Data with unequal variances were log-transformed, and the homogeneity of variances was verified before the completion of analyses. Hypergeometric testing was performed using the function phyper from the package *stats* (version 4.4.0). The RT-qPCR data did not meet the assumption of normality, therefore, a non-parametric Kruskal–Wallis test was performed in Prism (version 10.4.2) followed by a Dunn’s multiple comparisons test.

## Results

### The Transcriptomic Profile of Myometrium from Testosterone-treated Patients Resembles that of Myometrium from Fibroid Patients

**Fig. 1:**
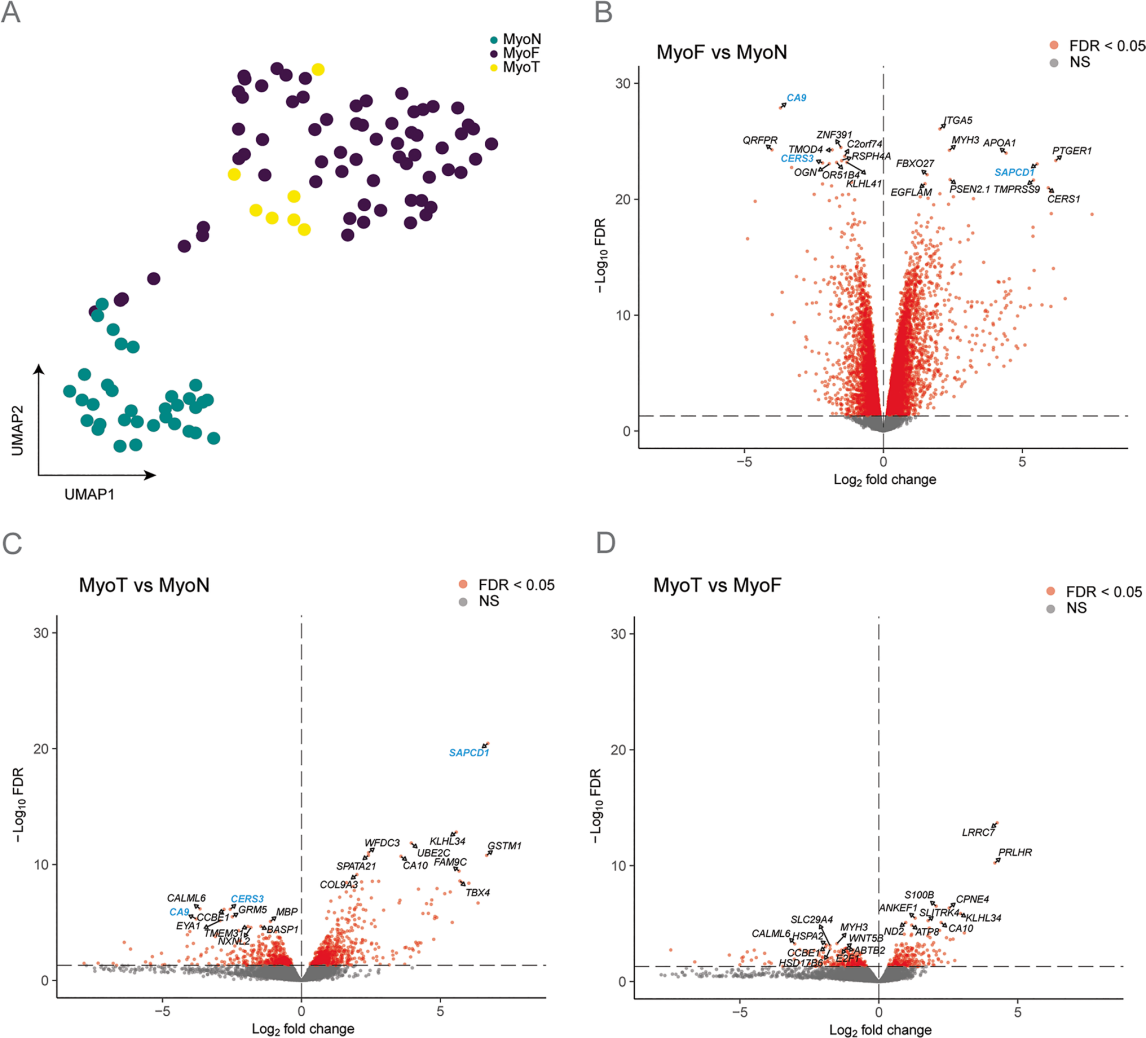
RNA-sequencing results of the different myometrial tissues. (**A)** Uniform Manifold Approximation and Projection plot after batch correction of all expressed genes shows separation by tissue type (MyoN n = 33, MyoF n = 66, MyoT n = 7). Each dot represents an individual sample. Volcano plots show the up- and down-regulated protein coding genes with a false discovery rate (FDR) p-value < 0.05 depicted as red dots in MyoF vs. MyoN **(B),** MyoT vs. MyoN **(C)** and MyoT vs. MyoF **(D)**. The top 10 up- and down-regulated differentially express protein coding genes for each comparison are annotated. The top genes in common between MyoF vs. MyoN and MyoT vs. MyoN are shown in blue

Bulk RNA-seq analysis of myometrium tissue from MyoN, MyoF and MyoT samples was first visualized by projection in a UMAP plot (Fig. 1A). Two main clusters were observed, one containing only MyoN samples and a second cluster comprising all MyoF and MyoT samples, along with three MyoN samples. Unsupervised hierarchical clustering of all expressed genes in myometrium samples reveals that the two primary branches largely separate MyoN samples from MyoF and MyoT samples (Figure S1A). Principal component (PC) regression analysis further demonstrates that MyoN samples are distinct from MyoF and MyoT samples along PC1, which accounts for 13.64% of the variance (Figure S1B).These results confirm our previous findings that MyoN and MyoF samples differ [14], and indicate that, at the transcriptomic level, MyoT samples are more similar to MyoF than to MyoN.

To further explore this finding, we compared the 3 tissue types, MyoN, MyoF, and MyoT to each other to identify DEGs (FDR < 0.05). Volcano plots from MyoF vs MyoN (Fig. 1B), MyoT vs MyoN (Fig. 1C), and MyoT vs MyoF (Fig. 1D) revealed 8,901 (3,901 down- and 5,000 up-regulated), 1,321 (570 down- and 751 up-regulated) and only 494 (265 down- and 229 up-regulated) protein coding genes, respectively (Supplementary Table 2, Supplementary Table 3 and Supplementary Table 4). Suppressor APC domain containing 1 (*SAPCD1*), Ceramide synthase 3 (*CERS3*), and Carbonic anhydrase 9 (*CA9*) were among the top 10 significantly regulated coding genes that were also differentially expressed in both MyoF and MyoT compared to MyoN samples.

### Common Dysregulation of Fibroid-associated Pathways and Genes in MyoF and MyoT Compared to MyoN

**Fig. 2:**
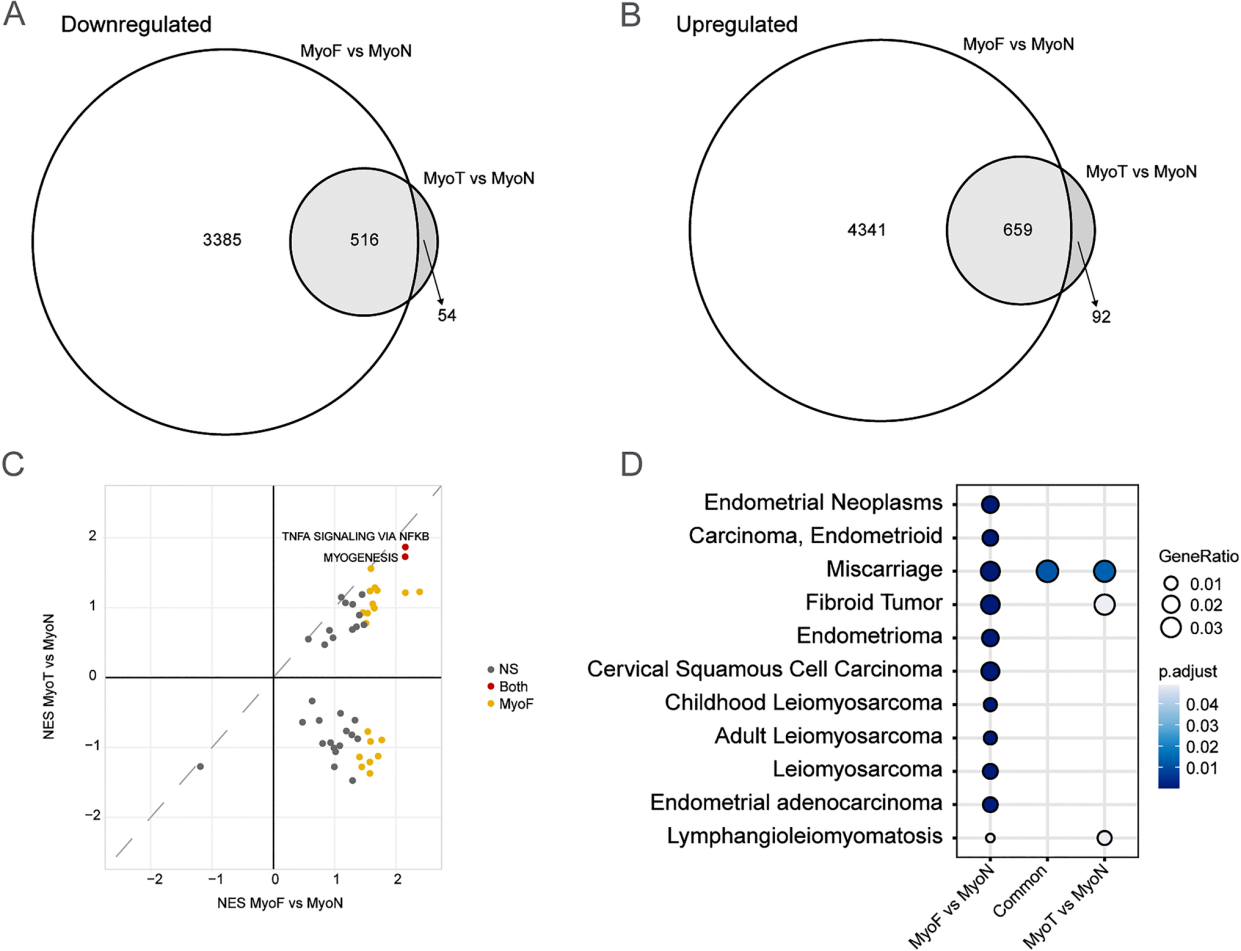
Transcriptomic similarities in MyoT and MyoF samples. Venn diagrams illustrate the overlapping down- **(A)** and up-regulated **(B)** differentially expressed protein coding genes between MyoF samples (n = 66) and MyoN samples (n = 33) and between MyoT samples (n = 7) and MyoN samples (n = 33). Hypergeometric testing reveals that the overlaps are significant, with p = 1.7 × 10^–253^ for the down-regulated genes and p = 9.3 × 10^–235^ for the up-regulated genes. Scatter plot of hallmark gene set enrichment analyses by normalized enrichment score (NES) shows gene sets increased in both comparisons in quadrant 1 **(C).** Top enriched Disease Gene Network from in DOSE (DGN) **(D)**. Gene ratio and significance level are shown by the size and color of each circle

**Fig. 3:**
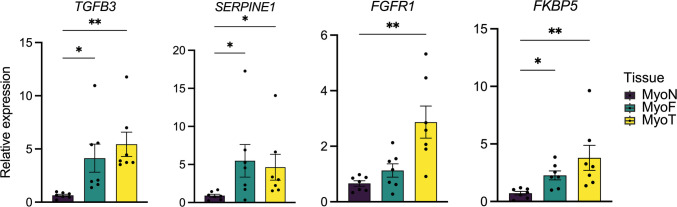
Expression of fibroid and androgen response related genes. Relative expression of dysregulated *TGFB3*, *SERPINE1*, *FGFR1*, and *FKBP5* mRNA by RT-qPCR in MyoN (n = 7), MyoF (n = 7) and MyoT samples (n = 7). *p < 0.05, **p < 0.01; by Kruskal–Wallis test

We next compared the differentially expressed protein-coding genes between MyoF vs. MyoN and MyoT vs. MyoN. Substantial and significant overlaps were observed, with 90.5% (n = 516) of down-regulated and 87.7% (n = 659) of up-regulated protein-coding genes in MyoT vs. MyoN compared to MyoF vs. MyoN (Fig. 2A and 2B). A scatter plot of the gene set enrichment analysis (GSEA) showed that two Hallmark pathways [28] are significantly enriched in both MyoT and MyoF when compared to MyoN, TNFα signaling via NFκB and myogenesis (Fig. 2C). Disease Gene Network (DGN) analysis of female reproductive track-related disease identified 46 and 4 significantly enriched disease gene sets [29] in MyoF vs. MyoN and MyoT vs. MyoN, respectively (Supplementary Table 5). Notably, fibroid tumor disease ranked as the top enriched disease in both MyoF vs. MyoN and MyoT vs. MyoN comparisons (Fig. 2D). Dysregulated fibroid associated gene expression was highlighted in boxplots grouped by tissue type (Fig. 3). Among the DEGs, transforming growth factor beta 3 (*TGFβ3*), serpin family E member 1 (*SERPINE1*) and fibroblast growth factor receptor 1 (*FGFR1*), which we have previously identified [14, 22], were also significantly upregulated in both MyoF and MyoT compared to MyoN tissues in this analysis (Supplementary Table 2, and Supplementary Table 4). We confirmed the dysregulation of *TGFB3*, *SERPINE1*, and *FGFR1* by quantitative RT-PCR (RT-qPCR) (Fig. 3). Additionally, RT-qPCR analysis of a known androgen-responsive gene, FKBP Prolyl Isomerase 5 (*FKBP5*), showed that it was significantly upregulated in MyoT and MyoF compared to MyoN, with no significant difference between MyoT and MyoF (Fig. 3).

### Methylation Status of Myometrium from Patient Treated with Testosterone Is Different from Myometrium from Free-fibroid Patient

**Fig. 4:**
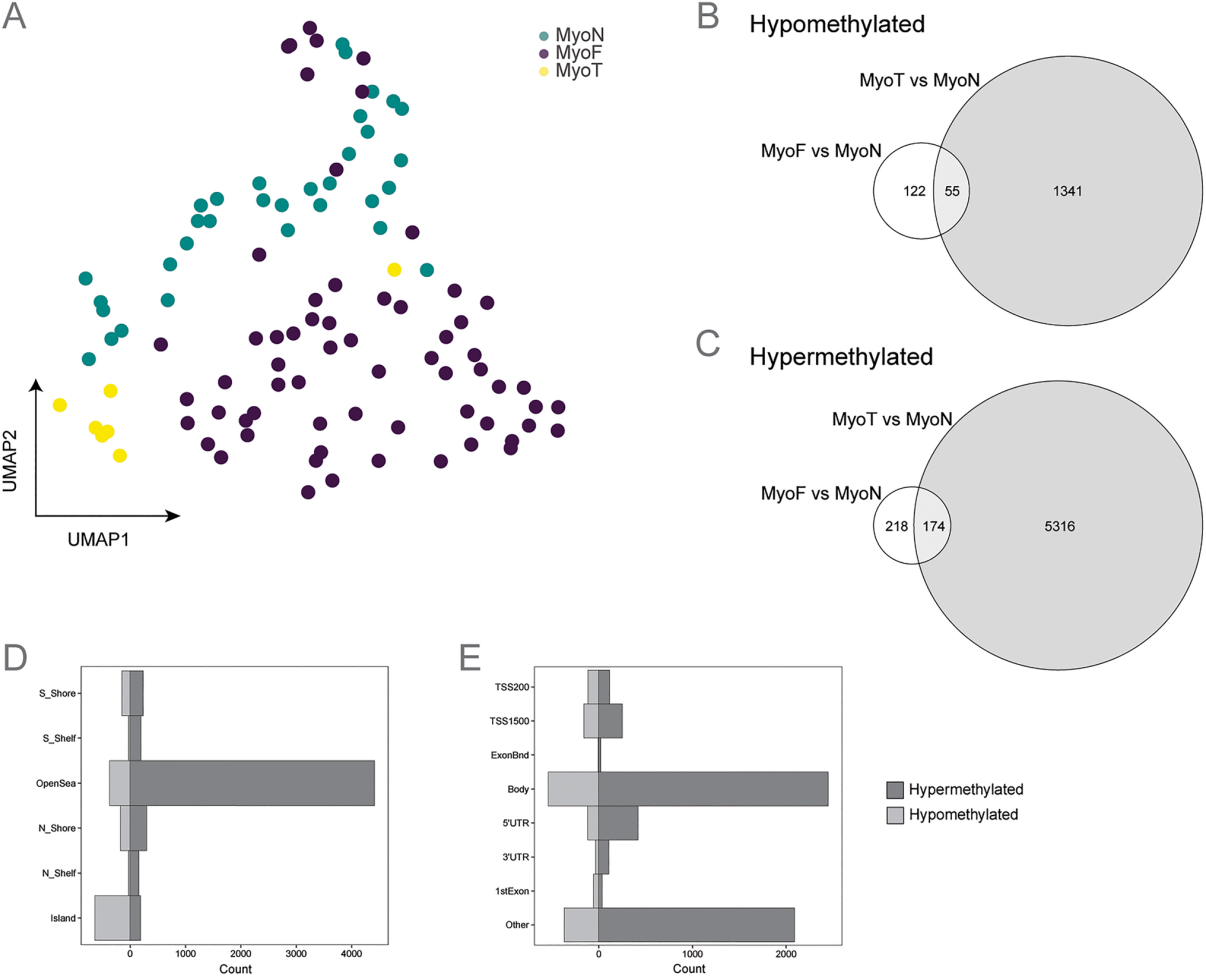
DNA methylation profiles of myometrium from fibroid-free patient (MyoN), fibroid patient (MyoF) and testosterone treated patient (MyoT). **(A)** Uniform Manifold Approximation and Projection plot after batch correction of beta values showing separation between tissue type (MyoN n = 33, MyoF n = 63, MyoT n = 7). Each dot represents an individual sample. Gene-associated differentially methylated loci (DMLs) for the MyoF vs MyoN and MyoT and MyoN samples are shown in a Venn diagram as hypomethylated **(B)** or hypermethylated **(C)**, with the overlap in the circles indicating shared DMLs. Hypergeometric testing of the overlaps in B and C revealed that they were statistically significant (p-values = 2.9 × 10^–91^ for the hypomethylated and 1.79 × 10^–215^ for the hypermethylated DMLs). Bi-directional barplot of the gene-associated DMLs positions **(D)** and their genomic contexts **(E)**. Barplots are shown as count of hypo- or hypermethylation CpG

Since hormone treatment for gender reassignment has been linked to changes in DNA methylation patterns over time [30], we compared the methylation status of MyoT samples with the MyoF and MyoN samples. Batch-corrected UMAP analysis of DNA methylation probes revealed only one cluster with each group of samples separated by tissue type (Fig. 4A). However, a PC analysis plot showed that the MyoN samples were mostly separated from the MyoF and MyoT samples by PC2 accounting for 7.01% of the variation (Figure S2B). As with the bulk RNA sequencing results, the DNA methylome of MyoT samples appears more similar to that of MyoF than to MyoN samples. Comparison of differentially methylated loci (DMLs) containing differentially methylated CpGs in MyoF and MyoT samples showed a higher total number of DMLs in MyoT than in MyoF when each is compared with MyoN. 6,886 DMLs (1,396 hypomethylated and 5,490 hypermethylated) were identified in MyoT vs. MyoN samples, compared to only 569 DMLs (177 hypomethylated and 392 hypermethylated) in MyoF vs. MyoN samples (Fig. 4B and 4 C, and Supplementary Table 6 and Supplementary Table 7). A significant overlap was observed in both comparisons, with 55 hypomethylated and 174 hypermethylated DMLs. We next classified the positions of DMLs (Fig. 4D) and analyzed their genomic context (Fig. 4E and Supplementary Fig. 2B). Compared to MyoN samples, most of hypomethylated DMLs in MyoT samples were located in CpG islands (46%) and OpenSea regions (26%), while the majority of hypermethylated DMLs were found in OpenSea regions (80%). Similar results were observed for the hypomethylated DMLs in MyoF compared to MyoN samples, however the hypermethylated DMLs were found in both CpG islands (39) and OpenSea regions (43%) (Figure S2C). In MyoT samples, both hypermethylated and hypomethylated DMLs were primarily located within gene bodies (hypermethylated = 45% and 3 hypomethylated = 39%) and in the intergenic regions (Other) with a higher number of hypermethylated DMLs (hypermethylated = 38% and 3 hypomethylated = 27%). Similar patterns were observed for hypomethylated DMLs in MyoF samples (Figure S2D).

## Discussion

Our study offers new insights into the impact of testosterone therapy on myometrial biology, revealing changes in transcriptomic and epigenetic profiles that mirror those seen in fibroid-associated myometrium (MyoF) compared with myometrium from patients without fibroids. By showing that testosterone-treated myometrial samples (MyoT) cluster with MyoF samples and separate from those without fibroids (MyoN) in both gene expression and DNA methylation patterns, we propose that testosterone may push myometrial tissue toward a more MyoF state. These findings suggest that the role of testosterone as a contributing factor in fibroid pathogenesis presents a promising avenue of investigation.

The substantial overlap in DEGs between MyoF vs. MyoN and MyoT vs. MyoN emphasizes the similarities between MyoT and MyoF tissues. Among the top dysregulated genes, several fibroid-associated genes, including *TGFβ3*, *SERPINE1*, and *FGFR1*, were significantly upregulated in both MyoF and MyoT compared to MyoN. These genes have well-documented roles in fibroid biology, such as promoting extracellular matrix deposition, and cell proliferation, further supporting testosterone’s potential contribution to fibroid development [14, 31–34]. Consistent with the transcriptomic shifts observed, we further validated the upregulation of a canonical androgen-responsive gene, *FKBP5*, in MyoT and MyoF samples relative to MyoN, with no significant difference between MyoT and MyoF. This shared upregulation supports the idea that androgen signaling pathways may be active in both testosterone-treated and fibroid-associated myometrium. *FKBP5*, a co-chaperone in glucocorticoid and androgen receptor signaling, was reported highly expressed in fibroids and may regulate genes involved in proliferation and apoptosis in leiomyoma cells [35, 36]. The concordant upregulation of these targets in both testosterone-treated and fibroid-associated myometrium supports a shared hormone-responsive state that could contribute to fibroid susceptibility. Pathway analysis revealed that hallmark pathways such as TNFα signaling via NFκB and myogenesis were significantly upregulated in MyoT compared to MyoN, mirroring the pathway enrichment observed in MyoF. This alignment underscores a shared molecular mechanism between testosterone exposure and fibroid-associated myometrium. Furthermore, Disease Gene Network analysis highlighted fibroid tumor disease as the top enriched condition in both MyoF vs. MyoN and MyoT vs. MyoN comparisons, reinforcing the functional relevance of these transcriptomic changes to fibroid biology. These findings align with epidemiological data linking hyperandrogenic conditions, such as polycystic ovary syndrome (PCOS) and obesity, to an increased risk of fibroids [13, 37, 38]. Indeed, the study from Wise et al*.* [13] suggested that women with PCOS, who often present with hyperandrogenism, were more likely to self-report having fibroids than those without PCOS. This association suggests that excess testosterone could exert long-term effects on myometrial cells, possibly contributing to fibroid initiation and growth. A meta-analysis of 24 studies involving over 325,000 participants found that individuals with obesity were more likely to report having fibroids. The study suggests that factors linked to obesity, including inflammation, insulin resistance, and metabolic syndrome, may contribute to fibroid risk [38]. Our findings align with these observations, as myometrium from the testosterone-treated patient showed transcriptomic and epigenetic changes that resemble MyoF, indicating that testosterone may actively contribute to a pre-fibroid or fibroid-prone state.

The methylation analysis showed that testosterone treatment produces a substantial number of differentially methylated loci (DMLs) in MyoT samples when compared to MyoN. Notably, PC analysis clustered MyoT and MyoF separately from MyoN by PC2, suggesting that testosterone induces epigenetic changes similar to those seen in fibroid-associated myometrium, even though the correlation is not as strong as observed in the RNA-sequencing results. The overlap of DMLs between MyoT and MyoF samples further supports the idea that testosterone may promote MyoF characteristics in myometrial tissue. CpG islands, key genomic regions with a high concentration of CpG sites, were notably affected, indicating that testosterone could drive lasting epigenetic changes in myometrium that contribute to MyoF-associated characteristics. Studies of hormone therapy, such as in gender-affirming treatment [39, 40] or menopausal hormone therapy [41], have consistently demonstrated that hormonal interventions can shift DNA methylation profiles over time​. For instance, research on transgender men has shown testosterone-induced methylation changes at hormone-sensitive genes, including estrogen receptor 2 gene (*ESR2*), suggesting that hormone therapies can lead to epigenetic modifications in pathways relevant to hormone responses [39]. Collectively, our findings suggest that testosterone may impact myometrial gene regulation via epigenetic alterations, even though a direct link between methylation changes and gene expression was not observed in this study. However, differential gene expression related to DNA methylation changes at distal DNA regions, such as enhancers, could still be an important mechanism by which testosterone therapy can influence myometrial cells. This underscores the need for further research into the epigenetic impact of testosterone on myometrium in conditions associated with elevated androgen levels.

One limitation of our study is that patients who received testosterone treatment were relatively young compared to the myometrium from fibroid or fibroid-free patients, potentially limiting the generalizability of our findings. Age-related differences in myometrial sensitivity and hormonal responses may affect the observed epigenetic and transcriptomic profiles, and these responses could differ in older patients who are also at risk for fibroid development [7]. Additionally, serum testosterone levels were not available for all patients in this study, limiting our ability to correlate tissue-specific responses directly with systemic androgen levels. Another limitation is that we were not able to assess whether the results could vary by different testosterone preparations used in treatment for gender dysphoria or whether those in the other groups were using prescription testosterone for other diagnoses. This gap restricts our interpretation of the direct impact of testosterone exposure and highlights the need for future studies with broader age groups and complete hormonal profiles for a more comprehensive analysis. It is also possible that testosterone indirectly affects myometrial cells by feedback inhibition of the hypothalamic-pituitary-ovarian axis, which inhibits follicular development and estrogen production by the ovary with the loss of pulsatile GnRH-regulated release of pituitary LH and FSH. Chronic postnatal treatment of female mice with androgens leads to decreased serum progesterone, acyclicity, ovarian defects, and increased uterine weight [42, 43]. Specific effects on myometrium were not reported and more studies are needed using in vivo and in vitro models to assess this possibility.

Our findings hold important implications for fibroid pathogenesis, especially in racial groups that tend to experience an earlier onset and more severe fibroid symptoms. Since testosterone levels vary widely and are often elevated in conditions such as PCOS and obesity, our results underscore the need to consider hormonal and metabolic factors in assessing fibroid risk. Future in vitro studies should investigate the effects of testosterone exposure on normal myometrial cells to determine whether androgen exposure drives the development of fibroid-like characteristics. Such studies could provide valuable insights into the mechanisms underlying fibroid development and inform the design of targeted therapeutic interventions.

In conclusion, this study suggests that testosterone may play an underappreciated role in fibroid pathogenesis by promoting genetic and epigenetic alterations in the myometrium that resemble those observed in fibroid tissues. Considering the association of elevated testosterone with conditions like PCOS and high BMI, testosterone may represent a modifiable risk factor in fibroid development. Understanding the mechanistic effects of testosterone on myometrial biology could open new avenues for preventive strategies and treatments for fibroids in high-risk populations.

## Supplementary Information

Below is the link to the electronic supplementary material.

Supplementary file1 (DOCX 27 KB)

Supplementary file2 (TSV 1831 KB)

Supplementary file3 (TSV 274 KB)

Supplementary file4 (TSV 103 KB)

Supplementary file5 (TSV 25 KB)

Supplementary file6 (CSV 1215 KB)

Supplementary file7 (CSV 96 KB)

Supplementary file8 (TIF 11426 KB)

Supplementary file9 (TIF 5976 KB)

## Declarations

These studies have been funded by grants from the Eunice Kennedy Shriver National Institute of Child Health and Human Development K99HD112539 and SRI/Bayer discovery innovation grant to ENP, and R01HD096259 to JMT.
