# Supplementary material for: The Human Myometrial Transcriptome and the DNA Methylome of Testosterone-treated Patients Resemble the Myometria from Fibroid Patients
Source: Reprod Sci. 2025 Jun 5;32(7):2223–32. doi: 10.1007/s43032-025-01893-9 (PMC12271253; doi:10.1007/s43032-025-01893-9)
Supplement: Supplementary file 1 — Supplementary file1 (DOCX 27 KB) [file 43032_2025_1893_MOESM1_ESM.docx]

| **Sample ID** | **Race** | **Age** | **Tissue type** | **[Total testosterone] ng/dL** |
| --- | --- | --- | --- | --- |
| GO537 | AA | NA | MyoF | NA |
| HF1 | AA | 38 | MyoF | NA |
| HF2 | C | 50 | MyoF | NA |
| HF4 | AA | 43 | MyoF | NA |
| HF8 | AA | 43 | MyoF | NA |
| HF9 | AA | 51 | MyoF | NA |
| HFB | A | 44 | MyoF | NA |
| HF10 | C | 45 | MyoF | NA |
| HF12 | AA | 49 | MyoF | NA |
| HF13 | AA | 44 | MyoF | NA |
| HF14 | AA | 38 | MyoF | NA |
| HF15 | AA | 40 | MyoF | NA |
| HF18 | AA | 48 | MyoF | NA |
| HF19 | AA | 49 | MyoF | NA |
| HF23 | AA | 49 | MyoF | NA |
| HF24 | AA | 51 | MyoF | NA |
| HF25 | Multi | 37 | MyoF | NA |
| HF27 | AA | 39 | MyoF | NA |
| HF28 | AA | 39 | MyoF | NA |
| HF29 | AA | 39 | MyoF | NA |
| HF33 | AA | 45 | MyoF | NA |
| MP61 | C | 59 | MyoF | NA |
| MP100 | C | 44 | MyoF | NA |
| MP111 | AA | 44 | MyoF | NA |
| MP136 | C | 45 | MyoF | NA |
| MP142 | C | 48 | MyoF | NA |
| MP278 | C | 40 | MyoF | NA |
| MP282 | AA | 39 | MyoF | NA |
| MP286 | H | 35 | MyoF | NA |
| MP291 | C | 48 | MyoF | NA |
| MP295 | AA | 45 | MyoF | NA |
| MP305 | C | 44 | MyoF | NA |
| MP306 | H | 49 | MyoF | NA |
| MP308 | C | 44 | MyoF | NA |
| MP313 | C | 46 | MyoF | NA |
| MP315 | C | 45 | MyoF | NA |
| MP317 | C | 37 | MyoF | NA |
| MP319 | C | 45 | MyoF | NA |
| MP321 | AA | 39 | MyoF | NA |
| MP322 | A | 42 | MyoF | NA |
| MP323 | C | 36 | MyoF | NA |
| MP330 | C | 37 | MyoF | NA |
| MP332 | C | 43 | MyoF | NA |
| MP342 | C | 52 | MyoF | NA |
| MP344 | AA | 45 | MyoF | NA |
| MP345 | C | 41 | MyoF | NA |
| MP346 | C | 50 | MyoF | NA |
| MP348 | AA | 45 | MyoF | NA |
| MP351 | C | 43 | MyoF | NA |
| MP353 | C | 45 | MyoF | NA |
| MP355 | C | 41 | MyoF | NA |
| MP359 | C | 46 | MyoF | NA |
| MP364 | C | 44 | MyoF | NA |
| MP366 | AA | 48 | MyoF | NA |
| MP369 | C | 42 | MyoF | NA |
| MP380 | AA | 45 | MyoF | NA |
| MP381 | AA | 51 | MyoF | NA |
| MP396 | C | 41 | MyoF | NA |
| MP416 | AA | 45 | MyoF | NA |
| MP423 | AA | 43 | MyoF | NA |
| MP456 | A | 45 | MyoF | NA |
| MP521 | C | 40 | MyoF | NA |
| MP541 | C | 52 | MyoF | NA |
| NW2 | AA | NA | MyoF | NA |
| NW206 | AA | NA | MyoF | NA |
| MP356 | C | 40 | MyoN | NA |
| MP358 | C | 47 | MyoN | NA |
| MP361 | C | 36 | MyoN | NA |
| MP362 | C | 41 | MyoN | NA |
| MP363 | C | 34 | MyoN | NA |
| MP365 | C | 43 | MyoN | NA |
| MP397 | C | 33 | MyoN | NA |
| MP398 | C | 36 | MyoN | NA |
| MP399 | C | 29 | MyoN | NA |
| MP402 | C | 43 | MyoN | NA |
| MP407 | C | 34 | MyoN | NA |
| MP408 | C | 41 | MyoN | NA |
| MP450 | C | 36 | MyoN | NA |
| MP451 | C | 31 | MyoN | NA |
| MP452 | C | 36 | MyoN | NA |
| MP459 | C | 31 | MyoN | NA |
| MP472 | C | 42 | MyoN | NA |
| MP473 | C | 34 | MyoN | NA |
| MP478 | C | 29 | MyoN | NA |
| MP479 | C | 31 | MyoN | NA |
| MP484 | C | 30 | MyoN | NA |
| MP485 | AA | 47 | MyoN | NA |
| MP490 | C | 39 | MyoN | NA |
| MP495 | C | 37 | MyoN | NA |
| MP496 | C | 42 | MyoN | NA |
| MP497 | C | 30 | MyoN | NA |
| MP499 | C | 31 | MyoN | NA |
| MP500 | C | 35 | MyoN | NA |
| MP502 | C | 42 | MyoN | NA |
| MP516 | C | 34 | MyoN | NA |
| MP542 | C | 23 | MyoT | NA |
| MP545 | C | 28 | MyoT | 127.8 |
| MP550 | C | 26 | MyoT | NA |
| MP551 | C | 24 | MyoT | 1137 |
| MP552 | AA | 32 | MyoN | NA |
| MP554 | C | 23 | MyoT | 35.4 |
| MP411 | C | 45 | MyoF | NA |
| MP506 | AA | 50 | MyoN | NA |
| MP561 | AA | 36 | MyoN | NA |
| MP562 | AA | 21 | MyoT | 48.8 |
| MP565 | C | 28 | MyoT | 211.7 |

**Supplementary table 1: Patient information**

C: Caucasian, AA: Black/African American, H: Hispanic, A: Asian, Multi: Multi-race, NA: Not available
