# Supplementary figures and images for: The Human Myometrial Transcriptome and the DNA Methylome of Testosterone-treated Patients Resemble the Myometria from Fibroid Patients
Source: Reprod Sci. 2025 Jun 5;32(7):2223–32. doi: 10.1007/s43032-025-01893-9 (PMC12271253; doi:10.1007/s43032-025-01893-9)

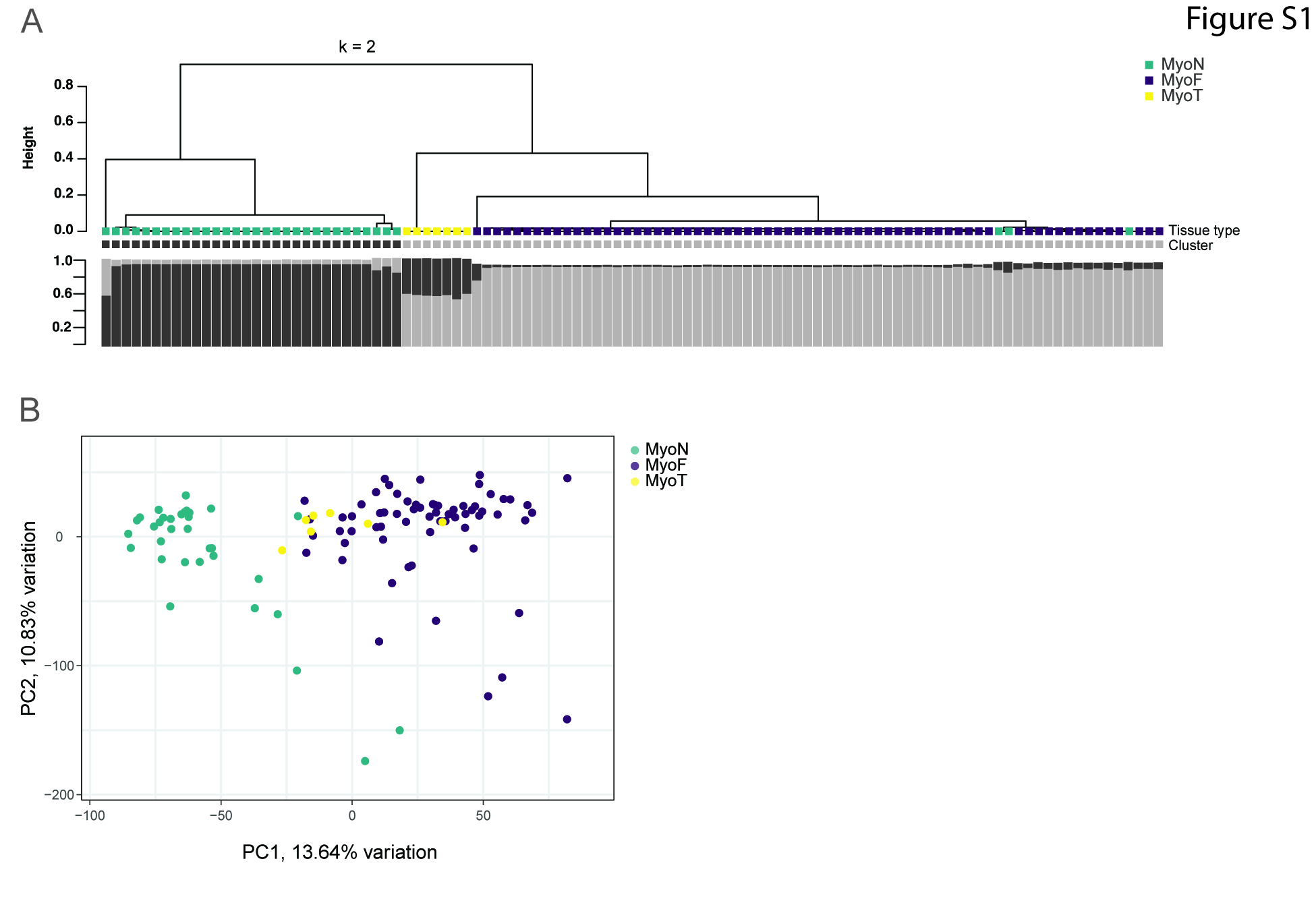

Supplement: Supplementary file 8 — Supplementary file8 (TIF 11426 KB) [file 43032_2025_1893_MOESM8_ESM.tif]

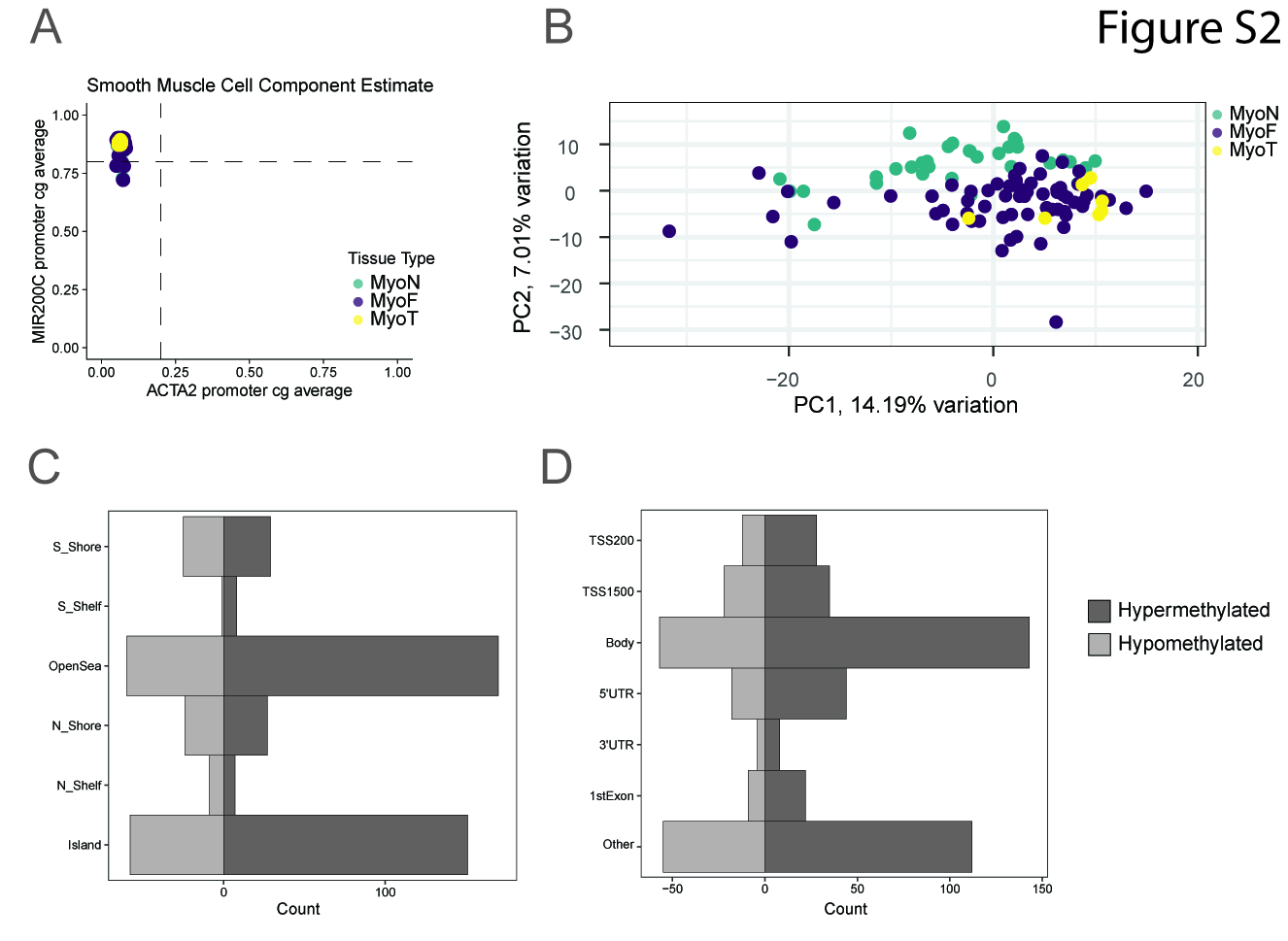

Supplement: Supplementary file 9 — Supplementary file9 (TIF 5976 KB) [file 43032_2025_1893_MOESM9_ESM.tif]
